# Implicit Rationing of Incretin Therapy in Diabetic Kidney Disease: A Call for Deliberate Allocation

**DOI:** 10.7759/cureus.115170

**Published:** 2026-08-25

**Authors:** Diego de Silva, Achille Peiris

**Affiliations:** 1 Nephrology, Balboa Nephrology Medical Group, San Diego, USA

**Keywords:** chronic kidney disease, drug pricing, drug rationing, glp-1 receptor agonists, healthcare equity, health policy, medication access, renal outcomes

## Abstract

Glucagon-like peptide-1 (GLP-1) receptor agonists have produced strong renal outcome data in patients with type 2 diabetes and chronic kidney disease (CKD), including a large randomized trial stopped early for benefit. Yet the patients with the best claim to these drugs are often the ones least able to obtain them. Supply and price are driven less by nephrologic risk than by a consumer market organized around elective weight loss, direct-to-consumer telehealth, and, until recently, a large compounding economy; at list price, cost-effectiveness analyses place these agents well above accepted value thresholds. This editorial argues that the resulting pattern of access amounts to rationing by default rather than by policy, and that nephrology has an obligation to name it and to press for allocation guided by clinical need. Whether these agents work in CKD is settled. The open question is who receives them once ability to pay, rather than risk of kidney failure, sets the order of the line. When access is constrained, we argue, priority should follow indication and kidney risk rather than purchasing power.

## Editorial

Introduction

Something has gone slightly sideways in the care of diabetic kidney disease over the past two years. We finally have a drug class that slows nephron loss in a measurable way [1], yet a surprising share of the clinical work around it has nothing to do with deciding to prescribe it. It has to do with getting the prescription filled: the prior authorization and the formulary exclusion. The patient who mentions, almost offhand, that the pharmacy wanted more than $1,000 for the month and that he left without it. Talk to the clinicians and coordinators who see these patients, and it stops sounding like the exception. It sounds like the rule.

Meanwhile, the same molecules have become some of the most visible products in American medicine, prescribed at volume for an indication that carries no risk of dialysis. The authors want to be careful here, because there is nothing wrong with that use on its own. Obesity is a disease, and treating it is real medicine. However, when a high-volume elective market runs into a supply and reimbursement system that cannot cover everyone, someone gets squeezed out, and it is not random who. The patient who loses tends to have advanced diabetic kidney disease, public insurance, and no slack in the monthly budget for a four-figure drug nobody is subsidizing. He is not being denied a lifestyle drug. He is losing time he cannot get back.

The science was the easy part

The FLOW trial showed that semaglutide cut a composite renal outcome in patients with type 2 diabetes and chronic kidney disease (CKD), and the benefit was large enough to stop the trial early [1]. Kidney Disease: Improving Global Outcomes (KDIGO) guidance now folds this class into the core of how we manage diabetes with kidney disease [2]. Nobody credible is still litigating the biology. That part is cleared.

What is not clear is the part where a patient actually obtains the drug. Cost-effectiveness work in the US puts these agents, at list price, well past the $100,000 to $150,000 per quality-adjusted life-year (QALY) most analyses use to define value; first-line use would clear that bar only if prices fell by roughly 70% for sodium-glucose cotransporter-2 (SGLT2) inhibitors and 90% for glucagon-like peptide-1 (GLP-1) receptor agonists [3]. Modeling from other systems lands in the same place, with SGLT2 inhibitors justifiable on cardiorenal grounds while GLP-1 receptor agonists stay hard to defend economically at current prices [4]. This leaves the tension nobody wants to state flatly. The drug works. The system, financed the way it is now, cannot give it to everyone who would benefit. Call it what it is: rationing.

Rationing nobody admits to

Every health system rations something. What differs is whether it happens on purpose, by criteria someone would defend in public, or by accident, through paperwork and price and whichever patient has the stamina to appeal a denial a third time. American incretin access is the accidental kind, and CKD patients come out of it worse in at least three ways.

Start with the payer logic, which runs backward. Coverage follows the glycemic indication and fights the weight-loss one, yet the strongest renal data come from a population defined by kidney risk that has little to do with how tightly a patient's glucose is controlled [1]. The patient whose real reason for needing the drug is holding onto the nephrons he still has gets judged against rules written for a different problem.

Then there is the cash market, which quietly sets a floor under the price. A large group willing to pay out of pocket for something non-urgent removes the one pressure that might otherwise drag prices toward the level the economists say we need [3]. Put bluntly, demand from people without kidney disease helps hold the price where people with kidney disease cannot reach it.

In addition, the compounding channel that used to take the edge off is now closed. When the Food and Drug Administration decided the semaglutide and tirzepatide shortages were over, it pulled the enforcement discretion that had let compounded versions circulate, and the cheaper parallel supply went with it [5]. The authors do not fault the safety logic; the quality concerns were real. However, the closure fell hardest on the patients least able to absorb branded pricing, including many with CKD, and the safety benefit and the access loss did not land on the same people.

What the field should say

The reflexive answer has been to lobby for broader coverage. That is fine as far as it goes, but on its own it does little, because widening coverage without touching price just moves the cost around and payers clamp down elsewhere [3]. Three moves would do more.

Nephrology should say plainly that when supply or affordability is genuinely tight, prioritizing by indication is the right thing to do. If a payer or a health system must pick, the patient with an estimated glomerular filtration rate (eGFR) of 35 mL/min/1.73 m² and albuminuria has a stronger claim than someone pursuing weight loss with no end-organ disease. The criteria are not mysterious. Reduced eGFR, meaningful albuminuria, a trajectory of decline, and the absence of an equally good alternative are the same variables we already use to grade kidney risk; the only new step is letting them shape access when access is short. The authors know how that lands. It sounds like a lecture about obesity, which it is not; obesity is a serious disease, and the authors would not argue otherwise. It is simply triage, the ordinary kind of medicine does whenever a resource runs short, and our squeamishness about saying so has handed the decision to the market. We do not have to settle this alone. We do have to be in the room, instead of reading about the outcome later.

We should also insist that renal benefit be priced and negotiated on its own, not bundled quietly into diabetes or obesity coverage. Keeping one patient off dialysis saves a large sum [4], and much of that saving accrues to a payer other than the one covering the drug during the years the damage is being averted. That mismatch is a choice, not a law of physics, and outcomes-based contracts or indication-specific pricing could correct it.

Additionally, we should be far more direct about who ends up on the losing side. Access built on cash, telehealth subscriptions, and employer benefit design tracks income, and income tracks the same lines along which kidney outcomes are already unequal. A drug class that could close that gap is, priced and marketed the way it is, on track to widen it. It is what you get when purchasing power decides who receives a drug that matters most to the people with the least of it.

None of this is as tidy as it sounds on the page. Indication-based prioritization is far easier to endorse than to operationalize; someone must set the threshold, adjudicate the borderline patient, and defend the line when it moves. The equity argument cuts more than one way too, since obesity itself concentrates among the disadvantaged, and a framework that deprioritizes weight-based use risks reproducing the inequities it means to correct. The authors do not think these objections defeat the case, but they change what it asks for. The claim is not that nephrology should draw the line. It is that a line is already being drawn, badly, by default, and that clinical judgment belongs somewhere in a process now governed by price alone.

Two versions of the next decade sit in front of us. In one, these drugs meaningfully cut how many people reach kidney failure. In the other, they become one more good therapy that mostly helps whoever can pay. The evidence has already picked a direction; what is left is price, coverage design, and whether the field will say the awkward part out loud. This is not unfamiliar ground. Nephrology's founding fights were about who got dialysis when there were not enough machines, and we worked through them by building committees, then a national program, then a settled conviction that ability to pay should not decide who lives.

The same candor is what this moment asks for, and it means something more specific than exhortation. Our professional societies could convene a working group on equitable access, put forward explicit criteria for prioritization when supply or affordability is constrained, and press payers for indication-specific pricing and outcomes-based contracts. It also means measuring the problem: we still lack good data on which CKD patients actually receive these drugs, and that gap is itself a kind of answer.

Not choosing is a choice too. This one deserves to be made on purpose.
